# Location of chlorogenic acid biosynthesis pathway and polyphenol oxidase genes in a new interspecific anchored linkage map of eggplant

**DOI:** 10.1186/s12870-014-0350-z

**Published:** 2014-12-10

**Authors:** Pietro Gramazio, Jaime Prohens, Mariola Plazas, Isabel Andújar, Francisco Javier Herraiz, Elena Castillo, Sandra Knapp, Rachel S Meyer, Santiago Vilanova

**Affiliations:** Instituto de Conservación y Mejora de la Agrodiversidad Valenciana, Universitat Politècnica de València, Camino de Vera 14, 46022 Valencia, Spain; Department of Life Sciences, Natural History Museum, Cromwell Road, London, SW7 5BD UK; Center for Genomics and Systems Biology, New York University, 12 Waverly Place, New York, NY 10003 USA; Center for Genomics and Systems Biology, New York University Abu Dhabi Research Institute, Abu Dhabi, United Arab Emirates

**Keywords:** Chlorogenic acid, Genetic map, Polyphenol oxidases, *Solanum incanum*, *Solanum melongena*, Synteny

## Abstract

**Background:**

Eggplant is a powerful source of polyphenols which seems to play a key role in the prevention of several human diseases, such as cancer and diabetes. Chlorogenic acid is the polyphenol most present in eggplant, comprising between the 70% and 90% of the total polyphenol content. Introduction of the high chlorogenic acid content of wild relatives, such as *S. incanum*, into eggplant varieties will be of great interest. A potential side effect of the increased level polyphenols could be a decrease on apparent quality due to browning caused by the polyphenol oxidase enzymes mediated oxidation of polyphenols. We report the development of a new interspecific *S. melongena* × *S. incanum* linkage map based on a first backcross generation (BC1) towards the cultivated *S. melongena* as a tool for introgressing *S. incanum* alleles involved in the biosynthesis of chlorogenic acid in the genetic background of *S. melongena.*

**Results:**

The interspecific genetic linkage map of eggplant developed in this work anchor the most informative previously published genetic maps of eggplant using common markers. The 91 BC1 plants of the mapping population were genotyped with 42 COSII, 99 SSRs, 88 AFLPs, 9 CAPS, 4 SNPs and one morphological polymorphic markers. Segregation marker data resulted in a map encompassing 1085 cM distributed in 12 linkage groups. Based on the syntheny with tomato, the candidate genes involved in the core chlorogenic acid synthesis pathway in eggplant (*PAL*, *C4H*, *4CL*, *HCT*, *C3′H*, *HQT*) as well as five polyphenol oxidase (*PPO1*, *PPO2*, *PPO3*, *PPO4*, *PPO5*) were mapped. Except for *4CL* and *HCT* chlorogenic acid genes were not linked. On the contrary, all *PPO* genes clustered together. Candidate genes important in domestication such as fruit shape (*OVATE*, *SISUN1*) and prickliness were also located.

**Conclusions:**

The achievements in location of candidate genes will allow the search of favorable alleles employing marker-assisted selection in order to develop new varieties with higher chlorogenic content alongside a lower polyphenol oxidase activity. This will result into an enhanced product showing a lower fruit flesh browning with improved human health properties.

**Electronic supplementary material:**

The online version of this article (doi:10.1186/s12870-014-0350-z) contains supplementary material, which is available to authorized users.

## Background

Eggplant (*Solanum melongena* L., Solanaceae; 2n = 2× = 24) ranks third in the genus *Solanum*, after potato and tomato, in total production and economic importance and is the most important Solanaceae crop native to the Old World [1]. The most nutritionally important bioactive constituents of the eggplant fruit are phenolics, which are responsible of the high antioxidant activity of eggplant [2-6]. The most abundant phenolics of eggplant are hydroxycinnamic acid (HCA) conjugates, which are synthesized by converting phenylalanine to cinnamic acid. Among HCA conjugates, chlorogenic acid (5-*O*-caffeoyl-quinic acid; CGA) constitutes between 70% to over 95% of the total phenolics content [7-10]. Growing interest in this compound is due to its many beneficial properties for the treatment for various metabolic and cardiovascular diseases and ailments. Several *in vitro* and *in vivo* experiments have shown that CGA has anti-oxidant, anti-inflammatory, analgesic, antipyretic, neuroprotective, cardioprotective, anti-carcinogenic, anti-microbial, hypotensive, anti-obesity and anti-diabetic activity [5,11,12]. Moreover, CGA is highly stable at high temperatures, and its bioavailability in eggplant increases, as compared to the raw product, after cooking [3].

Great diversity in the content of total phenolics and CGA has been observed in eggplant, due both to genetic and environmental factors [2,6,7,9,10,13]. Some close wild relatives of cultivated eggplant, such as *S. incanum* [14,15], have high levels of CGA [7,9,16]. *Solanum incanum* is native to northern Africa and the Middle East to Pakistan [15], and is a cross-compatible with *S. melongena* [1,7]. Therefore, *S. incanum* shows promise for use in breeding programs for developing new eggplant varieties with increased phenolic content [5].

**Figure 1:**
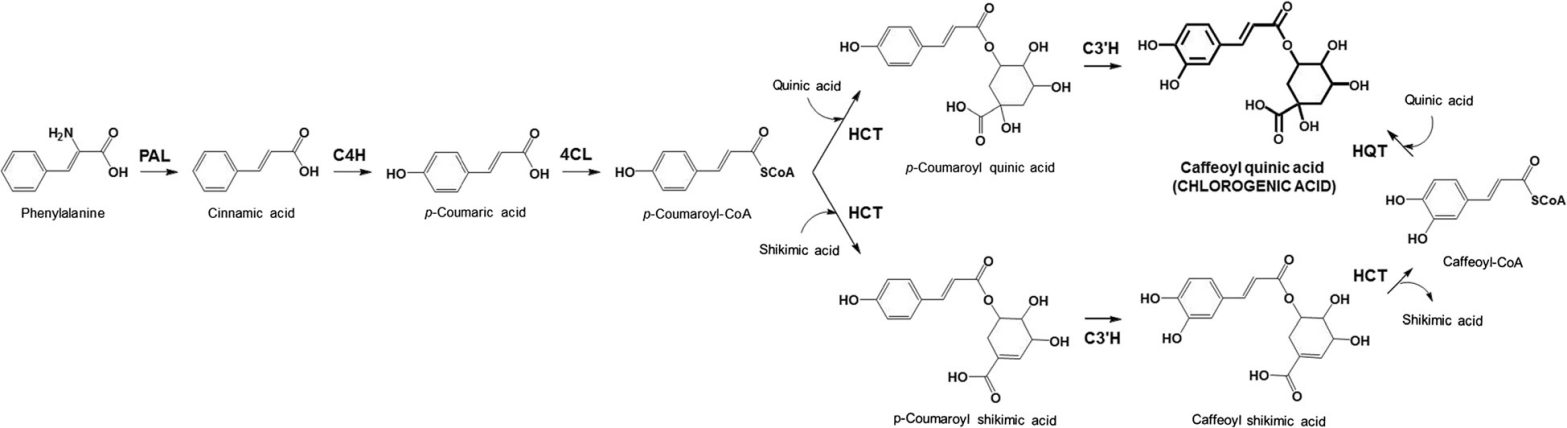
**Biochemical pathway for chlorogenic acid (CGA) synthesis in eggplant.** Enzymes involved in the CGA pathway are indicated: *PAL*, phenylalanine ammonia lyase; *C4H*, cinnamate 4-hydroxilase; *4CL*, 4-hydroxycinnamoyl-CoA ligase; *HCT*, hydroxycinnamoyl-coA shikimate/quinate hydroxycinnamoil transferase; *C3’H*,p-coumaroyl ester 3’-hydroxilase; *HQT*, hydroxycinnamoyl CoA quinate hydroxycinnamoyl transferase [21-24].

Raising the total phenolics content, however, may cause a negative effect on apparent quality of the fruit. When eggplant fruit flesh is cut, phenolics, mostly stored in vacuoles, become available to polyphenol oxidase enzymes (PPOs), which are present in chloroplasts. PPOs catalyse the oxidation of phenolics to quinones, which in turn, react non-enzymatically with oxygen in the air to give brown compounds, thus causing browning of fruit flesh [17]. Several authors have found differences in PPO activity between varieties of eggplant, which can lead to differences in the degree of browning in fruit flesh between varieties with similar content of total phenolics [18-20]. Molecular breeding for high CGA content and low PPO activity could contribute to developing improved cultivars with higher bioactive properties through a combination of high antioxidant activity and presenting a low degree of browning. For this purpose, a candidate gene approach shows promise, given that the genes involved in the CGA synthesis pathway, which include phenylalanine ammonia lyase, *PAL*; cinnamate 4-hydroxilase, *C4H*; 4-hydroxycinnamoyl-CoA ligase, *4CL*; hydroxycinnamoyl-coA shikimate/quinate hydroxycinnamoil transferase, *HCT*; *p*-coumaroyl ester 3’-hydroxilase, *C3’H*; and, hydroxycinnamoyl CoA quinate hydroxycinnamoyl transferase, *HQT*, (Figure 1), in addition to the PPO genes, are known [20-24].

Understanding of eggplant genome organization, which is of great relevance for molecular breeding, has lagged behind that for other solanaceous crops such as potato, tomato and pepper. Several linkage maps for eggplant have been developed. Nunome et al. [25] developed a first intraspecific linkage map in eggplant using RAPD and AFLP markers. Two improved versions of the Nunome et al. [25] map were developed by adding SSR markers [26,27]. Doganlar et al. [28] developed the first interspecific map using RFLP markers resulting from crossing *S. melongena* and *S. linnaeanum*. The resolution of this map was further improved by adding COSII and AFLP markers [29,30]. Barchi et al. [31] also developed an intraspecific mostly AFLP and SSR marker. Finally, an intraspecific saturated integrated map of *S. melongena* was developed by Fukuoka et al. [32] from two F_2_ populations in which SSR and SNP markers were mapped. Of the markers used by Fukuoka et al. [32], many were obtained from *Solanum* orthologous (SOL) gene sets from a multiple alignment between the unigenes of eggplant, tomato and potato.

Here we report the development of a new interspecific *S. melongena* × *S. incanum* linkage map with the aim of locating, and in the future introgressing, *S. incanum* alleles involved in the biosynthesis of CGA in the genetic background of *S. melongena*. In order to devise molecular tools for minimizing browning associated with high CGA levels, PPO genes were also targeted. This new map is anchored to the tomato genetic map and previous eggplant maps, which will facilitate molecular breeding in eggplant for high CGA content and reduced browning as well as other morphological traits of importance in eggplant breeding.

## Results

### Genetic map construction

**Figure 2:**
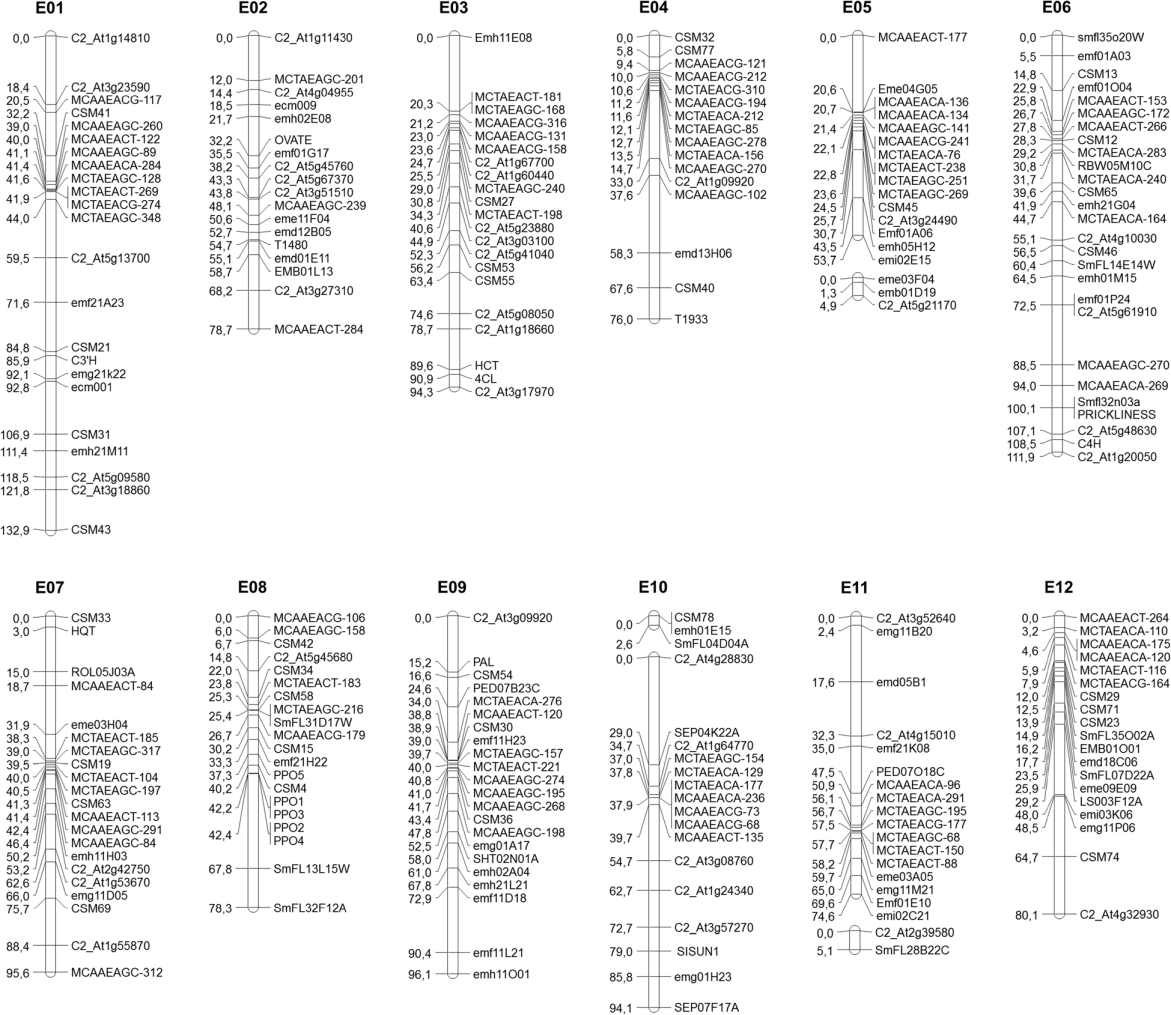
**The interespecific genetic linkage map SMIBC.** Linkage groups were denominated E01 to E12 in agreement with the denomination of linkage groups in other eggplant genetic maps [28,32,33]. The map distances, given in cM, are shown on the left side of the linkage groups. Marker names are shown on the right side.

The mapping population was genotyped with 243 molecular markers comprising 42 COSII, 99 SSRs, 88 AFLPs, nine CAPS, four SNPs and the morphological marker *PRICKLINESS*. Genotypic data generated a genetic linkage map that spans 1085 cM distributed in 12 major and three minor linkage groups (Figure 2). Synteny with maps of Wu et al. [29], Fukuoka et al. [32], Barchi et al. [33] and Tomato-EXPEN 2000 [34] anchored the three minor linkage group to the corresponding major linkage groups (E05, E10, E11). The linkage groups ranged in length between 58.6 cM (E05) and 132.9 cM (E01) (Table 1). The average genome-wide density was 4.46 cM, with linkage group E01 having the lowest average density (5.77 cM inter-locus separation), and E08 showing the highest density (3.19 cM inter-locus separation). The number of loci per linkage group was highest in E06 (27) and lowest in E04 (16). Segregation distortion was observed for 22.6% of the markers (Table 1). The linkage groups with greater distortion were E02, E03 and E09 with around half of their markers skewed. A clear distortion in favour of *S. incanum* was found in E03 and E09 whereas in E02 and E06, alleles for *S. melongena* were more abundant. In order to develop a strong framework map, only markers joined at LOD > 3 were selected and those that had a lower LOD were discarded to avoid errors in positioning.

**Table 1:** **Statistics of framework of SMIBC map**

	**Linkage groups**												
**Statistics**	**E01**	**E02**	**E03**	**E04**	**E05**	**E06**	**E07**	**E08**	**E09**	**E10**	**E11**	**E12**	**Total**
Length (cM)	132.9	78.7	94.3	76	58.6	111.9	101.7	78.3	96.1	96.7	79.7	80.1	1085.0
Number of markers	23	18	21	16	18	27	21	20	22	19	19	19	243
COSII	5	7	8	2	2	4	3	1	1	5	3	1	42
SSRs	8	7	4	4	7	13	8	9	12	6	9	12	99
AFLPs	9	3	7	10	9	8	9	5	8	7	7	6	88
CAPS	1	1	1	0	0	0	0	4	1	1	0	0	9
SNPs	0	0	1	0	0	1	1	1	0	0	0	0	4
Morphological	0	0	0	0	0	1	0	0	0	0	0	0	1
Average density (cM)	5.77	4.37	4.49	4.75	3.25	4.14	4.84	3.19	4.36	5.09	4.19	4.21	4.46
Gaps (>15 cM)	2	0	1	2	1	1	0	1	2	2	1	3	16
Skewed markers (*P* < 0,05)	0	9	11	0	3	5	2	1	11	7	2	4	55
Percentage skewed markers	0	50.0	52.3	0	16.6	18.5	9.5	5.0	50.0	36.8	10.5	21.0	22.6

The table shows the length in cM, the number of markers of each type, the average density in cM, the gaps larger than 15 cM and the skewed segregation for each linkage group. Linkage groups are designated as E01- E12.

### COSII analysis

**Figure 3:**
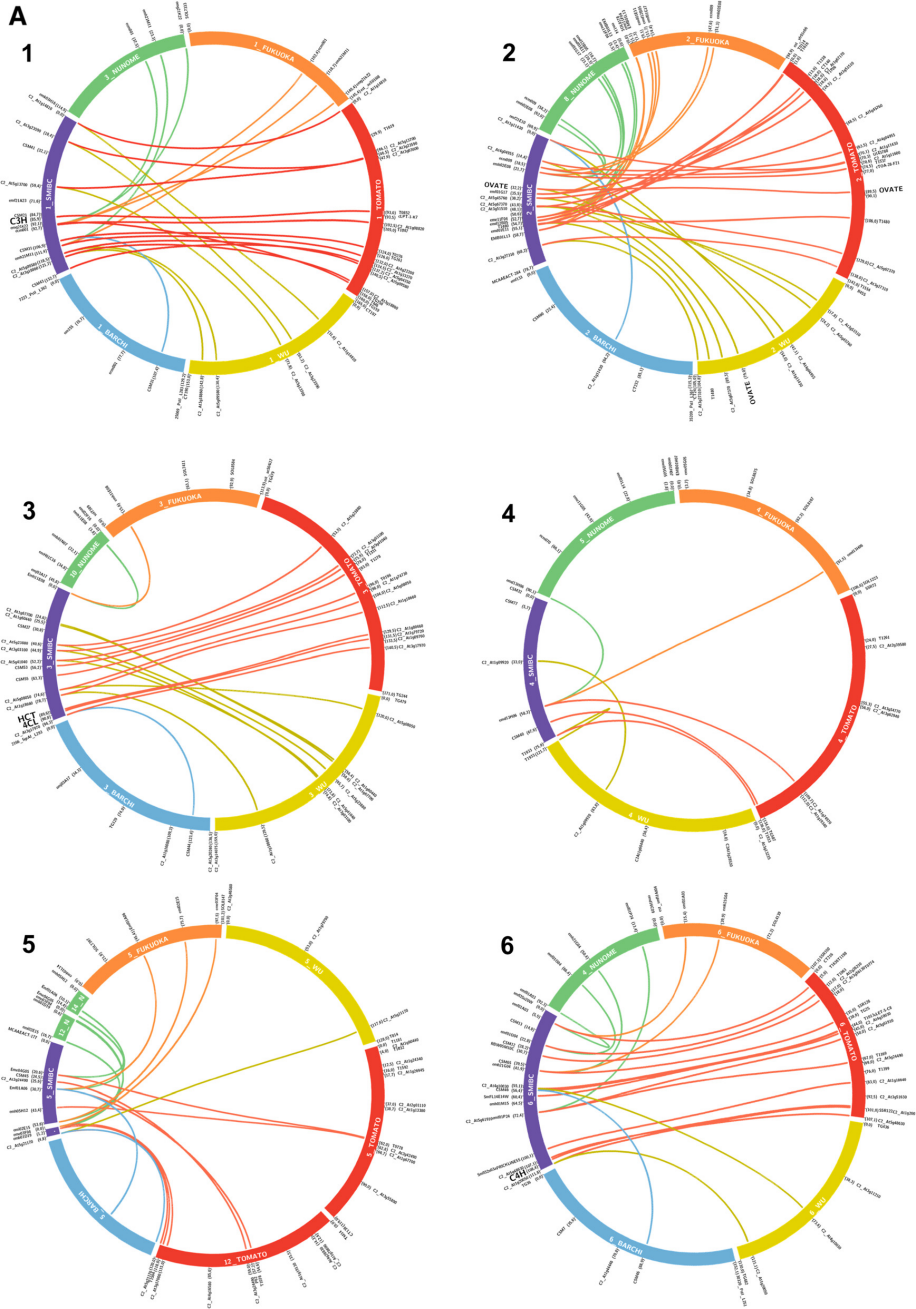
**Macro-synteny between SMIBC interspecific eggplant map, tomato and eggplant maps (linkage groups E01-E06).** Different colours were used for linkage groups and links to distinguish and anchor the maps. SMIBC interspecific eggplant map was depicted in purple, tomato EXPEN-2000 map [34] in red, Barchi et al. [33] eggplant map in blue, and Fukuoka et al. [32] eggplant map in orange, Nunome et al. [27] eggplant map in green, and Wu et al. [29] eggplant map in yellow. Inside of linkage groups in white are shown the corresponding map and number of each linkage group. On the external part of circular ideograms are indicated the markers name and their position. The candidate genes for chlorogenic acid (CGA) synthesis pathway and polyphenol oxidases (PPOs) are shown in bold letters.

**Figure 4:**
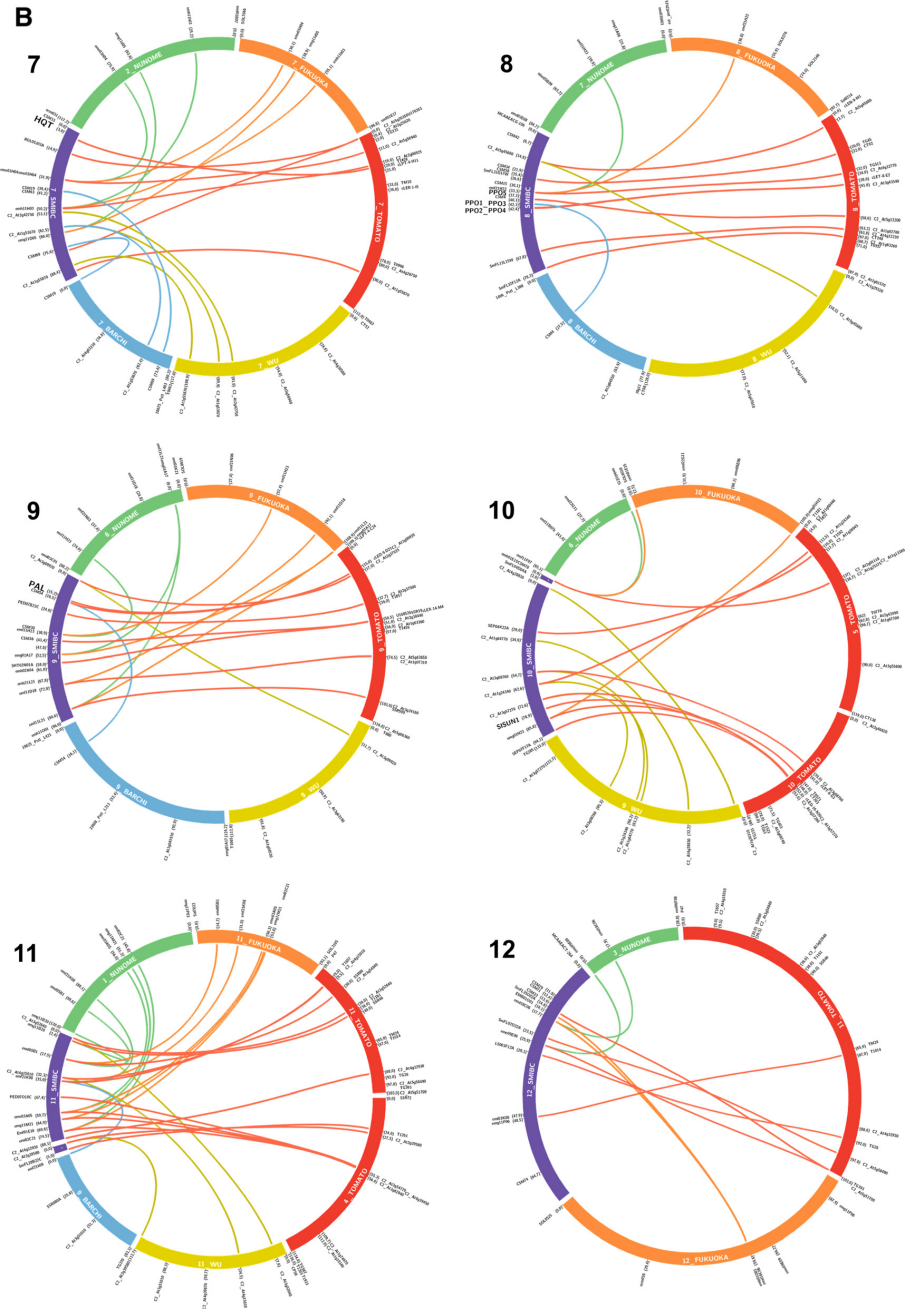
**Macro-synteny between SMIBC interspecific eggplant map, tomato and eggplant maps (linkage groups E07-E12).** Different colours were used for linkage groups and links to distinguish and anchor the maps. SMIBC interspecific eggplant map was depicted in purple, tomato EXPEN-2000 map [34] in red, Barchi et al. [33] eggplant map in blue, and Fukuoka et al. [32] eggplant map in orange, Nunome et al. [27] eggplant map in green, and Wu et al. [29] eggplant map in yellow. The corresponding number of each linkage group and map are shown inside the same. Markers name and their position are shown on the external part of circular ideograms. The candidate genes for chlorogenic acid (CGA) synthesis pathway and polyphenol oxidases (PPOs) are shown in bold letters.

A total of 35 (28.5%) out of the 123 COSII developed by Wu et al. [29] were polymorphic in our mapping population (Table 1). Seven other COSII markers were identified comparing the sequences of SSRs mapped with the tomato genome database (Sol Genomic Network). Six of them were EST-SSRs, obtained *in silico*, and one was a genomic SSR marker (Table 2). COSII markers allowed us to establish synteny with Wu et al. [29] eggplant map and with the Tomato EXPEN-2000 map [34] (Figures 3 and 4, Additional file 1: Figure S1) as well as with other members of the Asterid clade [35].

**Table 2:** **COSII markers identified in the SMIBC genetic map from CSM and EST-SSR markers based on sequence homology found after a BLASTN search on the SGN Cornell marker database** [70]

**SMIBC LG**	**Current marker name**	**Previous marker name**	**Type of SSR**
E03	C2_At3g17970	CSM44	genomic SSR
E04	C2_At1g09920	SmFL32K22A	EST-SSR
E05	C2_At3g24490	LS502D19A	EST-SSR
E06	C2_At5g61910	PLA03P07F	EST-SSR
E06	C2_At5g48630	ROT01I04F	EST-SSR
E08	C2_At5g45680	SmFL04A12A	EST-SSR
E12	C2_At4g32930	LS004H02A	EST-SSR

### SSRs analysis

A total of 99 SSR markers of different sources were mapped in the BC1 population (Table 1). One hundred and twenty-eight of the 254 EST-SSRs, obtained *in silico* using eggplant unigenes from the VegMarks database, showed homology with tomato unigenes (Sol Genomics Network). Of these, forty-seven were selected based on the theoretical position and screened for segregation in our parental (*S. incanum* and *S. melongena*) plants. Twenty of them (42.5%) showed polymorphism and were mapped. In addition, 71 genomic SSR markers from Nunome et al. [27], with an average of 4–5 per linkage group, were tested. Of these, 53 (74.6%) showed polymorphism and were positioned on the map. Most of them were also used by Fukuoka et al. [32] in the LWA2010 genetic integrated map, allowing the comparison between the two maps (Figures 3 and 4). Finally, 33 genomic SSRs (CSM markers) developed by Vilanova et al. [36] could be mapped. Seven of them were also used in Barchi et al. [33] in their genetic linkage map, enabling us to establish synteny (Figures 3 and 4).

### AFLPs analysis

A total of 116 AFLP polymorphic bands were produced from 12 AFLP primer combination combined with three MseI primers. A total of 88 AFLP markers were identified and mapped (Table 1), with an average of 9.6 polymorphic bands per primer pair. Scoring only AFLP bands present in *S. incanum* and absent in *S. melongena* in a backcrossing population where *S. incanum* is a donor parent had the advantage that AFLPs could be scored in a codominant manner.

### Mapping of CGA pathway genes

The six genes (Figure 1) involved in the core CGA synthesis pathway [21-24] were amplified and positioned in the SMIBC genetic map (Figure 2) based on the syntenic position with the Tomato EXPEN-2000 genetic linkage map [34] (Additional file 1: Figure S1, Additional file 2: Table S1).

#### PAL (phenylalanine ammonia lyase)

The search for phenylalanine ammonia lyase in SOL database give several orthologous gene sequence in tomato clustered in the same region of chromosome nine. A Blast of these orthologs in our local database allow to identify eggplant unigene OVS02A18A obtained by Fukuoka et al. [37]. A reciprocal Blast of OVS02A18A in SOL database shows the high homology with [SGN:Solyc09g007890.1.1] whose length is approximately 2.3 Kb and consist of two exons and one intron. In *S. melongena* and *S. incanum* parents a SNP (T/C) was found, after the amplification and sequencing of intron region, which was validated by restriction enzyme using a CAPS method (Additional file 3: Table S2). After genotyping the BC1 population the gene was mapped into the linkage group E09 at 15.2 cM from the linkage group end (Figure 2). The tomato orthologous gene is also positioned in the upper part on chromosome 9, between markers [SGN:CLED-9-D21] (15.0 cM) and [SGN:C2_At2g37025] (15.3 cM) and (Figure 4).

#### C4H (cinnamate 4-hydroxilase)

A search in the SOL database for the *C4H* gene in tomato yielded no results, however a *C4H* ortholog was found in potato [SGN:PGSC0003DMG402030469]. This gene in potato is approximately 3.7 kb and comprises three exons and two introns. Using this sequence it was possible to find the eggplant unigene SmFL27M04A and develop primers that amplify a region comprising the first intron. The amplicon in parents was sequenced and a SNP (A/G) was located and validated with high resolution melting (HRM) technique (Additional file 3: Table S2). The gene could be mapped on the bottom of linkage group E06 at 108.5 cM from the linkage group end (Figure 2). Synteny analysis reveals high co-linearity between linkage group 6 of eggplant and tomato. After a BLAST search using eggplant unigene SmFL27M04A, we found the tomato ortholog on linkage group 6 that corresponded to unigene [SGN-U590064]. Although this tomato gene, positioned in the Tomato EXPEN-2000 map between markers C2_At3g51630 (92.5 cM) and T1789 (95.0 cM) (Figure 3), was not annotated as *C4H*, it is certainly orthologous to *C4H*.

#### 4CL (4-hydroxycinnamoyl-CoA ligase)

Using a tomato *4CL* sequence [SGN:Solyc03g117870.2], which was 3.6 Kb with five exons and four introns, it was possible to find the eggplant unigene SmFL38N19A. Analysis of the amplified sequences of introns three and four, allowed us to detect a polymorphism (A/G) that was transformed in a CAP marker (Additional file 3: Table S2). The gene could be mapped in the lower part of linkage group E03 at 90.9 cM from the linkage group end (Figure 2). The orthologous gene in tomato is positioned in linkage group 3 between COSII markers [SGN:C2_At1g09760] (133.3 cM) and [SGN:C2_At1g16180] (133.5 cM) (Figure 3).

#### HCT (hydroxycinnamoyl-coA shikimate/quinate hydroxycinnamoyl transferase)

The eggplant unigene ROT01O23W was identified using a tomato ortholog [SGN:Solyc03g117600.2]. The gene in tomato (5.3 Kb) consists of three exons and two introns. The first intron was located in the 5’ UTR and there was insufficient data available for primers to be designed. The second intron was large (3.5 Kb) and we were unable to amplify it. No polymorphism between the two parental sequences was found after the amplification of first and the second exons. After a BLAST search with ROT01O23W unigene against database of contigs developed by Barchi et al. [38] we found a positive contig (22573:15433_PStI_67/3_NODE_1_L378; 15220_PStI_305E40_NODE_1_L282). On the basis of the contig sequence obtained, primers that partially amplify the second intron were developed. After the analysis of the sequenced amplicon, a SNP (T/A) was found and validated using HRM (Additional file 3: Table S2). The gene was mapped into the linkage group E03 at 89.6 cM very close to the *4CL* gene (90.9 cM) (Figure 2). In the Tomato EXPEN-2000 map both genes also appear close together (Figure 3), separated only by 159.8 Kb in the tomato physical map.

#### C3’H (p-coumaroyl ester 3’-hydroxilase)

An eggplant unigene YFR01I20A was identified using a tomato orthologous sequence [SGN:Solyc01g096670.2] which was approximately 3 Kb and contains three exons and two introns. Primers were developed to amplify the second intron of the gene where an Indel (TT) was found (Additional file 3: Table S2). Using this polymorphism as a CAP marker, the gene could be mapped in linkage group E01 at 85.9 cM from the linkage group end (Figure 2). Synteny study reveals that the linkage group 1 in eggplant and tomato are collinear except for minor position changes. The orthologous gene in tomato is located between the markers [SGN:T0852] (93.0 cM) and [SGN:cLPT-1-k7] (93.5 cM) (Figure 3).

#### HQT (hydroxycinnamoyl CoA quinate hydroxycinnamoyl transferase)

Using tomato ortholog sequence, the eggplant unigene YFR01H03A was identified. The sequence of the *HQT* gene in tomato [SGN:Solyc07g005760.2) was 3.7 Kb and shows two exons and one large intron of 2.1 kb. We tried to amplify the intron of the eggplant ortholog, but we were not able to obtain a clear band. The amplification of the two exons areas allowed us to detect a SNP (A/G) that was validated by HRM (Additional file 3: Table S2). The gene was located in the upper part of linkage group E07 at a distance of 3.0 cM from the first marker (Figure 2). The tomato orthologous gene is also positioned in the upper part of linkage group 7 between the markers [SGN:U176363] (0.2 cM) and [SGN:TG131] (2.0 cM) (Figure 4). Study of synteny based on 5 anchor points reveals certain collinearity between the two linkage groups although more anchor markers would be desirable.

### Mapping of PPO genes

From the alignment of the PPO sequences published by Shetty et al. [20], primers were designed in order to amplify six PPO genes in *S. melongena* and *S. incanum* parental plants. In order to shorten the names, in this paper the SmePPO genes described by Shetty et al. [20] are here simply termed PPO. All of them were selectively amplified and sequenced except for *PPO6*, which was amplified in *S. melongena* but not in *S. incanum.* SNPs polymorphisms were found in the other five PPO genes. CAPS could be developed for *PPO1* (C/A), *PPO2* (C/G), *PPO4* (G/A) and *PPO5* (G/A and T/G) while the SNP of *PPO3* (G/A) was validated by HRM (Additional file 3: Table S2). As we expected, based on synteny with Tomato EXPEN-2000 map [34] (Figure 4), all eggplant PPOs were mapped in SMIBC in the same genomic region in the linkage group E08, where *PPO1* and *PPO3* are situated at a distance of 42.2 cM, *PPO2* and *PPO4* at a distance of 42.4 cM, and *PPO5* at 37.3 cM from the linkage group end (Figure 2).

Synteny reveals that PPO orthologous genes in tomato are located in an area of 95.5 Kb, comprising the markers [SGN:TG624] (36.70 cM) and [SGN:ClET-8-E2] (38.0 cM) (Figure 4). Several comparisons between eggplant and tomato PPO were made to correctly assign orthologous PPO genes between the two species, but a clear identification was not reached, probably due to high sequence similarity among the PPO genes of each species.

### Mapping of other genes and traits of agronomic importance

The sequence of the *OVATE* gene [SGN:Solyc02g085500.2], which determines the conversion from round to pear-shaped fruit in tomato, corresponded to eggplant unigene SmFL28E15A. The gene was mapped into the linkage E02 at 32.2 cM from the linkage group end (Figure 2). In Wu et al. [29] map, *OVATE* gene was mapped at 79.0 cM from the linkage group end (Figure 3), and in tomato the orthologous gene is positioned into linkage group 2 at 89.50 cM (Figure 3). The *SlSUN1* gene in tomato [SGN:SGN-U569959], which controls elongated and pointed fruit shape, is positioned in chromosome 10, near the marker [SGN:C2_At3g10140] (52.80 cM) and shows a high identity with the eggplant contig (15541:35662_PstI_305E40_NODE_1_L250;36203_PStI_67/3_NODE_1_L230) [38]. The gene was mapped in SMIBC onto linkage group 10 at 79.0 cM from the linkage group end (Figure 2). The morphological marker *PRICKLINESS* could be mapped in linkage group E06 at 100.1 cM from the linkage group end (Figure 2).

### Synteny and orthologous candidate genes with other maps

Synteny using common molecular markers was established in order to develop a genetic linkage map taking advantage of the information provided by previous eggplant genetic linkage maps (Figures 3 and 4, Additional file 2: Table S1). The reference maps were the F_2_ intraspecific maps developed by Nunome et al. [27], the integrated map LWA2010 derived from two F_2_ linkage maps (LW2010 and AL2010) developed by Fukuoka et al. [32], the interspecific map *S. linnaeanum* (MM195) × *S. melongena* (MM738) developed by Wu at al. [29] and the F_2_ intraspecific maps developed by Barchi et al. [33]. In general a good conservation of marker location between SMIBC and the previous four eggplant maps was observed with the exception of six markers (Table 3). These markers show inconsistencies in position with Fukuoka et al. [32] map. In addition, macro-synteny between SMIBC and Tomato EXPEN-2000 map was established (Figures 3 and 4, Additional file 1: Figure S1, Additional file 2: Table S1). The syntenic relationship between these two maps was highly collinear, except for a few small parts of the genomes. In this way, a small part of the eggplant linkage group E03 was syntenic to tomato chromosome 5 (T05), and the same occurred between E05 and T05 and T12, E10 and T10 and T05, E11 and T11 and T04, and E12 with T11. These results are in agreement with the synteny observed by Wu et al. [29] among eggplant and tomato.

**Table 3:** **Markers with conflictive position according to the synteny between SMIBC interspecific eggplant map and Fukuoka et al.** [32] **LWA2010 eggplant genetic map**

**LG SMIBC map**	**Marker name**	**LG LWA2010 map**
E01	emf21A23	E08
E06	emh01M15	E03
E09	emh02A04	E11
E09	emh21L21	E07
E09	emh11O01	E03
E11	Emf01E10	E01

Linkage group (LG) in which the markers are positioned in SMIBC and LWA2010 maps are shown.

The highest number of anchoring points was observed with the Tomato EXPEN-2000 map, that shared 130 markers in common with SMIBC, varying from 15 (E02 and E06) to 4 (E04) per linkage genetic group, followed by the eggplant maps of Wu et al. [29] (42 anchoring points), Nunome et al. [27] (37), Fukuoka et al. [32] (32), and Barchi et al. [33] (12) for a total of 253 anchoring points. E02 was the linkage group with the highest number of links (38), while E04 had the fewest connections (8) (Table 4).

**Table 4:** **Statistics of macro-synteny between SMIBC map, Tomato EXPEN-2000 map developed by Fulton et al.** [34] **and eggplant genetic maps developed by Barchi et al.** [33]**, Fukuoka et al.**[32]**, Nunome et al.** [27]**, and Wu et al.** [29]

	**Linkage groups**												
**Genetic maps**	**E01**	**E02**	**E03**	**E04**	**E05**	**E06**	**E07**	**E08**	**E09**	**E10**	**E11**	**E12**	**Total**
Tomato EXPEN-2000	12	15	12	4	8	15	8	11	12	12	12	9	130
Barchi *et al*. (2012) [33]	1	1	1	0	2	1	3	1	1	0	1	0	12
Fukuoka *et al*. (2012) [32]	3	7	1	1	3	2	3	1	4	2	5	0	32
Nunome *et al*. (2009) [27]	3	7	1	1	5	4	3	1	3	1	6	2	37
Wu *et al*. (2009) [29]	5	8	7	2	1	2	3	1	1	5	3	4	42
Total	24	38	22	8	19	24	20	15	21	20	27	15	253

Number of shared markers with eggplant linkage groups, indicated as E01-E12, are indicated.

## Discussion

Although notable efforts have been made recently to better understand the structure and organization of the eggplant genome, the available genomic information is still very limited when compared to other major Solanaceae crops such as tomato, potato and pepper. Our interspecific map spread 1085 cM, and the 15 linkage groups we have found could be traced back to 12 chromosomes through the use of markers shared with other previously published maps. The SMIBC was the first map that connects all the most informative previously published genetic maps for eggplant, allowing us to exploit the information generated by these maps (Figures 3 and 4). A total of 130 anchoring points connect SMIBC and Tomato EXPEN-2000, many of which are COSII, enabling us to establish synteny between the two genomes (Figures 3 and 4, Additional file 1: Figure S1, Additional file 2: Table S1). This allowed us to confirm the position of all candidate genes according to orthologous gene location in tomato. The syntenic relationships between these two maps are in agreement with those observed by Doganlar et al. [28], Wu et al. [29], and Fukuoka et al. [32] and provide information on the genome evolution of both crops.

A general problem in the construction of genetic maps is that different types of molecular markers tend to map in a specific regions of the genome [27,32,39,40]. Our use of a wide variety of molecular markers including those used in previous eggplant genetic maps [27,29,32,33] allowed us to achieve better representation of the eggplant genome, which is important for effective molecular breeding and synteny studies in the future.

Distorted markers occurred in almost all linkage groups, except for E01 and E04. Segregation distortion in mapping populations is a well-documented phenomenon in different crops [41,42]. Similar distortion levels to those we observed have been reported by Doganlar et al. [28] in their interspecific map between *S. melongena* and *S. linnaeanum*, with 16% of markers being skewed towards the one or the other parent.

CGA is the major phenolic compound present in eggplant, which is one of the crops with highest content in hydroxycinnamic acids [5,8-10]. Until now, attempts to improve the CGA content in eggplant have been conducted in a classical way through hybridization and selection of materials with high CGA content [4,5,7]. The results of these programs have been positive, but due to moderate heritability (*H*^*2*^) of the CGA content trait and total phenolics in general, genetic advances have been limited [7,43,44]. Although these studies indicate that phenotypic selection to obtain materials with higher contents of CGA is possible, it would be desirable to apply marker-assisted selection (MAS) to improve the efficiency of selection for this trait.

The candidate gene (CG) approach has been successful in both animal and plant genetics [45]. The involvement of CGs in CGA biosynthesis pathway has been studied in plant species. The PAL gene catalyses one of the first steps of the phenylpropanoid pathway, that produces hundreds of specialized metabolic products including lignins, flavonoids, alkaloids and many other important phenolics in plants [46]. In *Populus trichocarpa*, five *PAL* genes have been described that are involved in wood formation [47], and in raspberry (*Rubus idaeus*) two *PAL* genes have been identified: *RiPAL1* associated with early fruit ripening events while *RiPAL2* involved with larger flower and fruit development [48]. Candidate genes involved in phenylpropanoid pathway have also been mapped in other plants like apple [49] and artichoke [50-52]. These are examples showing that homologs can have different functions, and paralogs can play largely different roles in CGA or lignin synthesis. Even within the Solanaceae, different genes in the pathway are strongly associated with the abundance of CGA. *HQT* seems to be the most important contributor to CGA synthesis in tomato and *Nicotiana benthamiana* [24], while *HCT* and *C3’H* are the most relevant in potato [53,54].

In this work we were able to locate all the genes involved in the core CGA pathway in eggplant. The positions of the genes are in agreement with those expected based on synteny with tomato. With the exception of *HCT* and *4CL*, which are co-situated at a genetic distance of 1.3 cM in the linkage group E03, all of the rest of the genes of the biosynthesis pathway of CGA are situated in separate linkage groups. This has important implications for breeding and for strategies based on pyramiding of favourable alleles introgressed from *S. incanum* in the genetic background of eggplant.

A potential problem for developing materials with higher CGA content is their oxidation mediated by polyphenol oxidase (PPO), which leads to the browning of the fruit flesh, reducing apparent fruit quality [5,18]. A positive phenotypic linear correlation was found in eggplant between the degree of fruit flesh browning and total content in phenolic and CGA content [4,43].

Several studies in other crops associated quantitative trait loci (QTL) for enzymatic browning with PPO alleles and these alleles were then used as markers for this trait [55,56]. On the other hand, silencing of PPO genes result in a reduced browning reaction as has been shown in potato and apple [57,58].

The studies of PPO genes suggest that in plants they form a gene family with high homology, usually have no introns and normally are present in several copies. For instance, tomato has seven PPOs, potato has six PPOs and soybean has eleven PPOs [56,59-61]. These features have complicated the search of polymorphisms between PPOs because amplification of a single isoform is difficult. In this study we were able to detect polymorphism in all PPO genes with the exception of *PPO6* (amplified in *S. melongena* but not in *S. incanum*) leading us to speculate that in *S. incanum* this PPO gene is absent or presents substantial changes with respect to *S. melongena*. As in tomato [60], eggplant PPOs were mapped very closely to each other in linkage group E08; this confirms the existence of a cluster also in eggplant (Figure 2), although a correct assignment using synteny was not possible between eggplant and tomato orthologs, due to high sequence similarity among the PPO genes of each species. In eggplant PPO genes identity varying between 72% and 95% at nucleotide level and between 62% and 92% at amino acid level [20].

Since both consumers and industry prefer eggplant varieties with white flesh and a low degree of browning, future varieties with high phenolic content should also be bred to have limited browning [43]. Accordingly, the search for allelic variants for increasing CGA content should be conducted in concert with a search for allelic variants decreasing PPOs. In eggplant, several authors have found differences in PPO activity between varieties [18-20] which suggest mining the biodiversity of this crop and its relatives can lead to discovering desirable alleles useful to produce varieties with low browning in fruit flesh regardless of the total phenolic abundance. In addition, the study of selection sweeps at these loci due to the culturally distinctive organoleptic preferences, would be of interest to understand the history of evolution of eggplant. A reduction in browning is quite probably a significant domestication trait in eggplant and many other species.

In the present study an interspecific genetic linkage map was developed to better understanding the genetics of CGA content and fruit flesh browning in eggplant through the mapping of candidate genes involved in these processes. This was assisted by the use of synteny of the orthologous genes in tomato. The use of *S. incanum* in this work and in breeding programs is not accidental, as it is a close relative of eggplant [14,15] and produces fully fertile hybrids with regular meiosis [7,14]. *Solanum incanum* has characteristics of great interest for plant breeders, such as bacterial and fungal resistance, including to *Fusarium oxysporum*, and tolerance to various abiotic stresses, especially drought [7,62]. Recent discoveries have shown that *S. incanum* is a powerful source of variation for phenolic content, as its total phenolic content is several times higher than that of *S. melongena* [7,9]. *Solanum incanum* also exhibits absence of anthocyanins, presence of prickles and subovoid green fruits [7].

Building an interspecific genetic map from the backcross generation of two species that differ in many traits has two important advantages. First of all, in this manner the problem of low intraspecific polymorphism in eggplant [28,29,32,63] is partially overcome. Secondly, this BC1 population can be used as the starting point to develop a set of introgression lines of *S. melongena*, with *S. incanum* as the donor parent. Once obtained, these lines will be useful to understand and dissect candidate gene roles in CGA accumulation and browning, to detect QTLs and genes involved in the synthesis of other polyphenols present in *S. incanum* (mainly *N*-(E)-caffeoylputrescine, 3-*O*-Malonyl-5-*O*-(E)-caffeoylquinic acid and 5-*O*-Malonyl-4-*O*-(E)-caffeoylquinic acid) [7], as well as many other morphological and physiological traits of interest for breeding or for crop evolution and domestication studies. Candidate genes for traits thought to be important in domestication such as fruit shape and *PRICKLINESS* are mapped in SMIBC in agreement with their positions based on previous QTL studies in eggplant and in syntenic regions of tomato [64,65], also highlighting the common occurrence of parallelism in loci causing domestication phenotypes between species.

## Conclusion

Despite its economic importance few genomic and biotechnological resources exist for eggplant. Eggplant contains high quantities of phenolics, in particular of CGA, a secondary metabolite with many properties beneficial for human-health and which in the last years is attracting a lot of attention from the scientific community. We report the development of a new interspecific map in eggplant using different types of markers. We have mapped the six genes involved in CGA synthesis pathway on five linkage groups, and determined that the PPO genes cluster together on LG8. This new map is anchored to the tomato genetic map as well as to four previous genetic maps of eggplant, and therefore constitutes an important tool for exploiting synteny and for molecular breeding in eggplant. The molecular tools we have developed will be of interest for eggplant breeding programmes, in particular those aimed at using marker assisted selection for improved CGA content combined with low browning effects. These tools and results, together with the recently published eggplant genome sequence [66] will greatly facilitate the development of a new generation of eggplant varieties with dramatically enhanced bioactive properties that can significantly contribute to better human nutrition.

## Methods

### Plant materials

**Figure 5:**
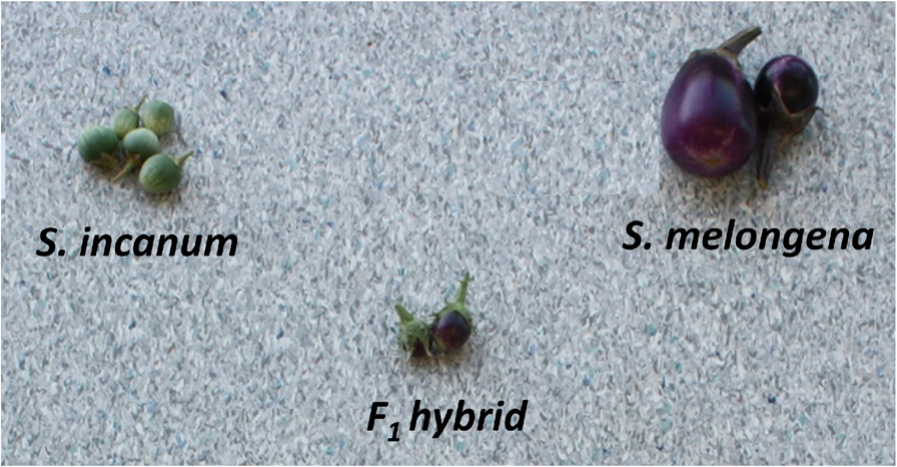
**Parentals and F1 hybrid used to generate the BC1 mapping population used to develop the SMIBC genetic map.**

Parentals for developing the interspecific genetic linkage map (denominated SMIBC, making reference to *S. melongena* and *S. incanum* [SMI] and backcross [BC] mapping population) were *S. melongena* accession AN-S-26 and *S. incanum* accession MM577 (Figure 5). The F_1_ hybrid was obtained using AN-S-26 as a female parent and MM577 as male parent. After that, the F1 hybrid was backcrossed (as a female) to the *S. melongena* AN-S-26 parental to obtain a BC1 population. BC1 seeds were germinated and 91 BC1 plants were randomly selected for the development of the genetic linkage map.

### Development of the SMIBC genetic map

#### DNA extraction

Total genomic DNA was isolated from leaves of the parental, F1 and BC1 plants according to the CTAB procedure [67]. DNA quality was checked in 1% agarose gels. DNA concentrations were measured with a Nanodrop ND-1000 spectrophotometer (Thermo Scientific, Wilmington, USA).

#### COSII markers

A total of 123 Universal Primers of COSII markers [68], previously mapped in an interspecific cross between *S. linnaeanum* and *S. melongena* [29], were screened in parental and F1 plants, and in the BC1 mapping population. Amplicons were analyzed on an ABI PRISM 3100-Avant DNA genetic analyzer (Applied Biosystems, Foster City, California). Amplicon sequences were examined with Sequence Scanner v1.0 (Applied Biosystems) software and aligned using the CAPS Designer software (http://solgenomics.net/) in order to find polymorphisms between the parental species. Restriction enzymes were used to cut in the polymorphic regions using the CAPS method (Cleaved Amplified Polymorphic Sequence). Alternatively, the High Resolution Melting (HRM) technique on a LightCycler 480 Real-Time PCR (Roche Diagnostics, Meylan, France) was used.

#### SSR markers

Three sources of SSR markers were used: a) EST-SSRs developed ‘*in silico*’ specifically for this study, b) genomic SSRs developed by Nunome et al. [27], and c) genomic SSRs developed using a genomic library [36]. Development of new SSR markers *in silico* was performed using 16,000 eggplant unigene sequences obtained from the VegMarks database (http://vegmarks.nivot.affrc.go.jp/VegMarks/jsp/index.jsp). The identification of perfect di- and tri-nucleotide motifs with a repeat of ≥6 times were identified using the software SciRoKo [69]. This allowed us the identification of 254 ESTs-SSRs, which were subjected to a BLASTN search of the SGN Cornell unigene database [70] (http://solgenomics.net/) against the tomato genome database. SSRs were validated in a 2-3% agarose gel when the differences in the bands sizes bands allowed polymorphisms in samples to be distinguished by eye. When this was not possible, the detection of polymorphisms was carried out by: a) capillary electrophoresis in polyacrylamide gel on an LI-COR 4300 DNA Analysis System combining fragment size and fluorophores IRD700 and IRD800 in a multiplex reaction, or b) by labeling forward primers with the fluorochromes FAM, VIC, NED, and PET. PCR products were diluted in formamide and analyzed on an automated DNA sequencer ABI PRISM 3100-Avant with a GeneScan-600LIZ (Applied Biosystems) size standard. The data were analyzed using the GeneScan software to obtain the electropherograms and polymorphisms were analyzed with Genotyper DNA Fragment Analysis software.

#### AFLP markers

Genomic DNA was digested with a two enzymes combination, EcoRI and MseI [71]. Then EcoRI and MseI adapters were ligated using an AFLP Core Reagent (Invitrogen, Carlsbad, CA) following the manufacturer’s instructions. A total of 12 AFLP primer combinations, generated by four EcoRI primers (EcoAGC, EcoACT, EcoACA, EcoACG) combined with three MseI primers (MseCAA, MseCTA, MseCAC) were used. A pre-amplification PCR reaction was executed with primers based on the adapter sequences with one additional selective nucleotide at the 3’ end of each primer (EcoA + MseC). Using an aliquot of the PCR product, a selective amplification reaction was performed with Eco and Mse primers combination with three additional selective nucleotides at the 3’ end of each primer. Each 5’ end of the EcoRI primers was labelled with different fluorescent dyes (PET, FAM, NED, and VIC). AFLP fragment analysis was performed on an automatic capillary eletrophoresis sequencer ABI PRISM 3100-Avant (Applied Biosystems, Foster City, CA). The data were analysed using GeneScan (Applied Biosystems) and Genotyper DNA Fragment Analysis (Applied Biosytems) software as above. Only AFLP bands that were present in in *S. incanum* and absent in *S. melongena* parental lines were scored.

#### *Sequence search for candidate genes and* in silico *comparison*

Orthologous sequences of candidate genes involved in CGA synthesis pathway were obtained from tomato and potato, by BLASTN searching in the SGN database. These sequences were used to find orthologous unigenes in an eggplant local unigene database, developed with the BioEdit software [72] (http://www.mbio.ncsu.edu/bioedit/bioedit.html). This local database contains the 16,245 unigenes published by Fukuoka et al. [37], available in the VegMarks webpage (http://vegmarks.nivot.affrc.go.jp). In addition, an eggplant local contigs database was made employing contigs developed by Barchi et al. [38]. The sequences of PPO genes published by Shetty et al. [20] were obtained from the NCBI database (http://www.ncbi.nlm.nih.gov/).

Intron/exon structure of the unigenes was detected by comparison with tomato sequences. Subsequently primers were designed with Primer3 software [73] to amplify preferentially introns in order to have more probabilities to find polymorphism. The amplification of genes was done according to the following protocol: denaturalization at 94°C for 5 min, 35 cycles at 94°C for 30 s, annealing at 55°C for 1 min, extension at 72°C for 2 min, and a final extension at 72°C for 10 min. In case of nonspecific amplifications, additional tests with higher annealing temperatures and/or lower MgCl_2_ concentrations were carried out.

The amplification products were purified and sequenced with an automatic ABI PRISM3100-Avant sequencer. Results were analysed with Sequence Scanner v1.0 (Applied Biosystems) software. The program Blast2Seq (NCBI) was used to compare the parental sequences to detect SNPs. SNPs found were transformed in CAPS markers with CAPS Designer software. In the cases in which no enzymes were available, primers to detect SNPs with High Resolution Melting (HRM) were developed. Synteny was studied between the SMIBC and the Tomato-EXPEN 2000 map [34].

#### Additional markers

The sequences and markers developed for *SlSUN1* and *OVATE*, which are candidate genes involved in fruit shape [74], were obtained as described above. The morphological marker *PRICKLINESS* was developed by phenotyping the plants and taking data on the presence or absence of stem prickles.

### Linkage analysis and map construction

The mapping population was genotyped and χ^2^ test (χ^2^ value ≤ χ^2^ α = 0.05) were performed to check if individual markers segregated following Mendelian ratios. Linkage analysis was carried out using Joinmap v4.0 software [75] to construct the map with settings LOD threshold of 3.0 and maximum recombination fraction θ = 0.4. Kosambi mapping function [76] was used to convert recombination units into genetic distance (cM).

#### Macro-synteny between SMIBC and other genetic linkage maps

The synteny of SMIBC map with other eggplant genetic linkage maps was established using shared markers [27,29,32,33]. The macro-synteny of SMIBC with the Tomato EXPEN-2000 [34] was carried out mainly using COSII markers developed by Wu et al. [68]. Some SMIBC SSR markers were positioned based on reciprocal best-hit relationships using BLASTN search against the “Tomato gene models CDS (ITAG release 2.30)” sequence database. Synteny was visualized using the Circos software [77].

### Availability of supporting data

All the data supporting our result are included in the article and in the Additional files.

## Additional files

Additional file 1: Figure S1. Image of macro-synteny established between SMIBC interspecific eggplant genetic map and Tomato-EXPEN 2000 genetic map. Additional file 2: Table S1. Data of the macro-synteny established between SMIBC interspecific eggplant genetic map, tomato and other eggplant genetic maps. Additional file 3: Table S2. Data of molecular markers mapped in SMIBC interspecific eggplant genetic map.
