# Supplementary figures and images for: Location of chlorogenic acid biosynthesis pathway and polyphenol oxidase genes in a new interspecific anchored linkage map of eggplant
Source: BMC Plant Biol. 2014 Dec 10;14:350. doi: 10.1186/s12870-014-0350-z (PMC4279458; doi:10.1186/s12870-014-0350-z)

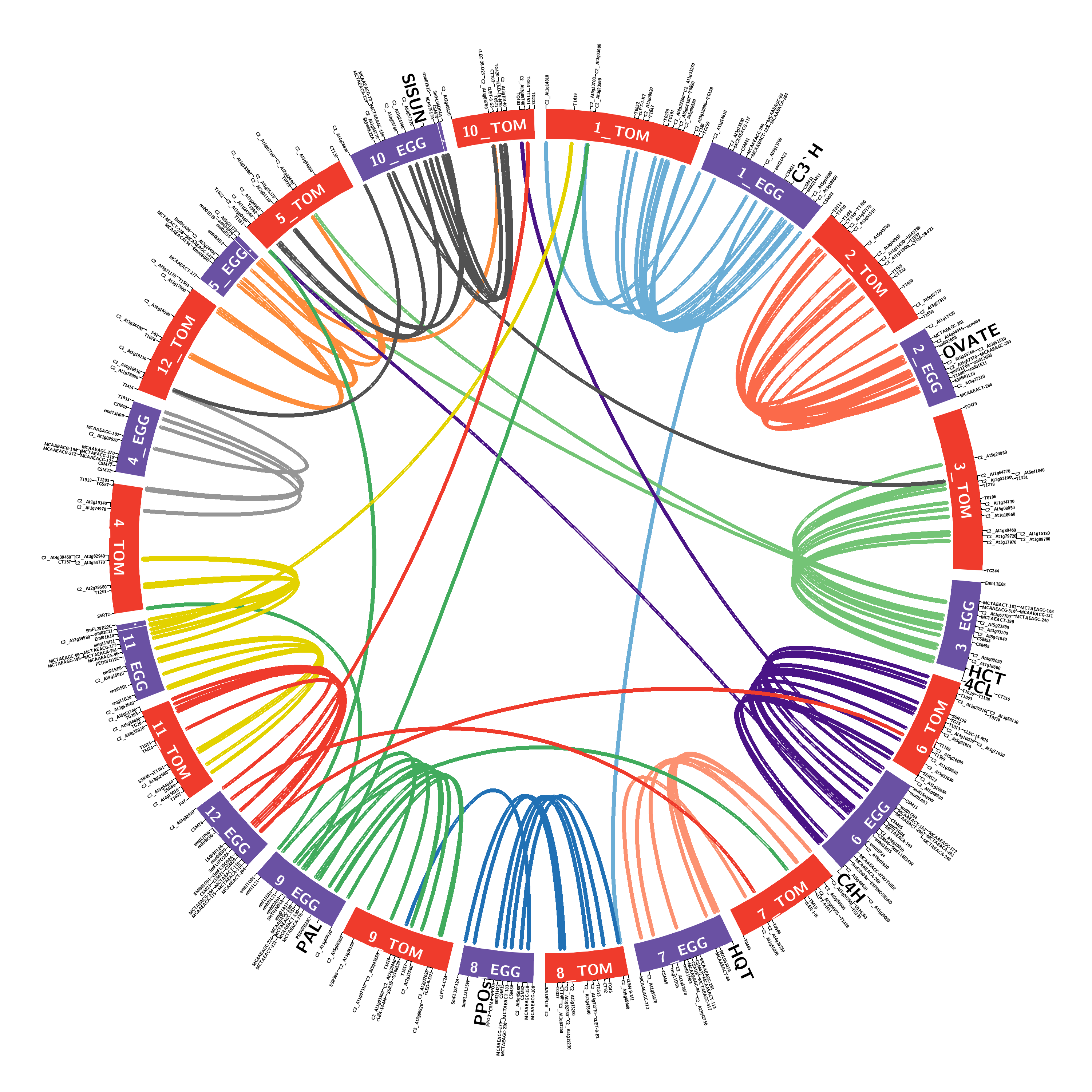

Supplement: Additional file 1: Figure S1. — Image of macro-synteny established between SMIBC interspecific eggplant genetic map and Tomato-EXPEN 2000 genetic map. [file 12870_2014_350_MOESM1_ESM.png]
